# What is the impact of mother’s bed incline on episodes of decreased oxygen saturation in healthy newborns in skin-to-skin contact after delivery: Study protocol for a randomized controlled trial

**DOI:** 10.1186/s13063-019-3256-0

**Published:** 2019-03-20

**Authors:** Jesús Rodríguez López, Nadia Raquel García Lara, María López Maestro, Javier De la Cruz Bértolo, José Carlos Martínez Ávila, Máximo Vento, Ana Parra Llorca, Isabel Izquierdo Macián, Adelina Pellicer, Natalia Marín Huarte, Izaskun Asla Elorriaga, Lourdes Román Echevarría, Cristina Copons Fernández, Ana Martín Ancel, Fernando Cabañas, Óscar García Algar, Carmen Rosa Pallás Alonso

**Affiliations:** 1 Neonatology Department, 12 de Octubre, University Hospital, Avenida de Córdoba s/n, 28041 Madrid, Spain; 2 Neonatology Department, La Fe University Hospital, Avenida de Fernando Abril Martorell, 106, 46026 València, Spain; 3 Neonatology Department, La Paz University Hospital, Paseo de la Castellana, 261, 28046 Madrid, Spain; 4 Neonatology Department, Cruces University Hospital, Plaza de Cruces, S/N, 48903 Baracaldo, Vizcaya Spain; 5 Neonatology Department, Vall d’Hebron University Hospital, Passeig de la Vall d’Hebron, 119-129, 08035 Barcelona, Spain; 6 Neonatology Department, San Joan de Déu University Hospital, Passeig de Sant Joan de Déu, 2, 08950 Esplugues de Llobregat, Barcelona, Spain; 7 Neonatology Department, Quironsalud Madrid University Hospital, Calle Diego de Velázquez, 1, 28223 Pozuelo de Alarcón, Madrid, Spain; 8 Maternal, Fetal and Neonatal Department, Hospital Sant Joan de Déu- Clínic University Hospital, Carrer de Villarroel, 170, 08036 Barcelona, Spain

**Keywords:** Pulse oximetry, Desaturation, Bradycardia, Tachycardia, Early skin-to-skin contact, Apparent life-threatening events, Sudden death, Angle of inclination, Full-term newborns

## Abstract

**Background:**

Early mother–child skin-to-skin contact (SSC) in the first 2 h postpartum is highly beneficial for both mother and child. However, cases have been reported of newborns who have experienced apparently life-threatening events (ALTEs) or sudden death during this procedure. The causes of these events are unknown. Newborn’s prone position could influence the onset of these events but there is very little evidence to support any recommendation. We hypothesize that newborns’ breathing obstruction episodes increase as mothers lie more horizontally.

The main objective of this study is to compare the occurrence of desaturation and bradycardia episodes as a function of mother’s bed incline.

The study is designed as a randomized, controlled, assessor blind, multicenter, superiority trial with two parallel groups and 1:1 allocation ratio.

**Methods:**

The study participants will be full-term healthy mother–newborn dyads from ten hospitals in Spain. Participants will be randomly assigned to one of two study arms defined by mother’s bed inclination (45° or 15°). The planned sample size is 5866. Centralized permuted blocks randomization and assessor blinding will be implemented.

The newborns will be monitored remotely with pulse oximetry, from 10 min to 2 h after delivery. We established SO_2_ and heart rate (HR) limit alarms, as well as an action protocol in the event of alarm activation.

The primary outcome is the number of healthy newborns who undergo episodes of SO_2_ ≤ 90%. Secondary outcomes are the mean SO_2_ level, the number of newborns who experience episodes of SO_2_ ≤ 85%, the time to SSC discontinuation due to abnormal SO_2_ or HR, and episodes of HR < 111 beats per minute (bpm) or > 180 bpm. Subgroups and pooled analysis will be performed to identify if breast-feeding and mother and child positions favor the occurrence of desaturation or bradycardia episodes.

**Discussion:**

A simple intervention such as modifying mother’s bed angle of inclination while in SSC with her child during the first 2 h postpartum could favor newborn’s hemodynamic and respiratory stabilization and thus contribute to reducing the onset of ALTEs.

**Trial registration:**

ClinicalTrials.gov, NCT02585492. Registered on 22nd October 2015.

**Protocol version:**

2 (30th June 2015).

**Electronic supplementary material:**

The online version of this article (10.1186/s13063-019-3256-0) contains supplementary material, which is available to authorized users.

## Introduction

### Background and rationale

Placing newborns in skin-to-skin contact (SSC) with their mother’s abdomen immediately after birth is currently recommended. This intervention has been recommended by the Baby Friendly Hospital Initiative [1] and the American Academy of Pediatrics [2] and is listed in Australian [3], Canadian [4], and British [5] guidelines. In Spain, this intervention is recommended by, among others, the Spanish Ministry of Health [6].

SSC promotes the establishment of an affective link between parents and child, a crucial part of the child’s survival and emotional development [7, 8]. SSC also improves the mother’s perceptions of her child, her maternal skills, and the newborn’s physiological stability [9–11]. The practice promotes the start and duration of maternal lactation, prolonging it by a mean of 43 days.

Coinciding with the widespread use of this intervention, however, cases have been reported of newborns who have experienced apparent life-threatening events (ALTEs) or sudden death in the first 2 h postpartum. These newborns had a gestational age ≥ 37 weeks, were born vaginally, and were apparently healthy [12–16]. These events have been reported in various countries [16–27]. Although there are still no appropriate registries, the global incidence rate appears to vary between 2.5 and 3.2/100,000 births, and the mortality rate varies between 0.8 and 1.8/100,000 births [16, 20]. The children who survive these events frequently experience severe neurological sequela [14, 15, 18, 19, 24–26].

The cause of these episodes is unknown [12, 13, 16, 17, 21, 22, 24], although the first 2 h after delivery are a critical period for newborns. A number of authors consider that newborns are more vulnerable to stressful hypoxic factors during this period due to airway obstructions (a condition that could arise when lying in decubitus prone on the mother’s body or breastfeeding) [12, 14, 16, 17, 19, 21, 25–27]. For other authors, these are newborns with prenatal impairment not initially manifested [20]. A number of authors have suggested that these events could be explained by a predominance of vagal tone during breastfeeding [13, 26]. These events have been observed more frequently in primiparous women, those who are tired or sleepy after delivery and those who were not monitored properly during SSC or while breastfeeding [12–17, 19–22, 24, 26, 27].

Due to the low incidence of ALTEs or sudden deaths in the first 2 h of life in SSC, it is very difficult to conduct prospective studies that enable the assessment of the efficacy of an intervention to prevent the ALTEs and sudden deaths. However, the fact that these events occur during the 2 h after delivery, a period in which most newborns are in a healthcare center, makes it feasible to design studies that evaluate the effect of various interventions on certain biological parameters that can be continuously monitored, such as SO_2_ and HR. Instability in either of these two parameters could precede these devastating events.

We found no information in the reviewed literature on the influence of the angle at which the mother’s bed is inclined while in SSC on SO_2_ and HR outcomes. Only a case study by Rodríguez-Alarcón et al. [27] recommends that the mother sit at 30° to 45° above the horizontal with a pillow behind the head but provides no information from any study to justify this recommendation. Given that the newborn’s prone position and the presence of circumstances that favor asphyxial episodes could influence the onset of these events, we could postulate that the more the mother is inclined, the more chances there are that the newborn will be in a situation that obstructs breathing during this critical period of life.

We therefore decided to conduct a randomized, multicenter clinical trial to compare the number of healthy newborns who experience episodes of SO_2_ ≤ 90% during the first 2 h after birth while in SSC with their mother while she is sitting up at a 45° or 15° angle above the horizontal plane of the bed.

### Objectives

The main objective is to compare the proportion of healthy newborns who undergo at least one episode of SO_2_ ≤ 90% between the two groups.

As secondary objectives, we will compare.

- the mean SO_2_ level
- the proportion of newborns who experience at least one episode of SO_2_ ≤ 85%
- the rate at which SSC is interrupted due to abnormal SO_2_ or HR
- episodes of HR < 111 beats per minute (bpm) or > 180 bpm

An additional secondary objective will be to identify positions for the mother and newborn that favor desaturation or bradycardia episodes.

### Trial design

The study is designed as a randomized, controlled, assessor blind, multicenter, superiority trial with two parallel groups and 1:1 allocation ratio.

The study has been approved by the Clinical Research Ethics Committee of the coordinating center (Hospital 12 de Octubre) and of each participating center. Additional file 1 contains the list of SPIRIT confirmation for the study.

## Methods

### Study setting

This study emerged from the research group setting of the Spanish Collaborative Research Network for Maternal and Child Health (SAMID Network; reference RD12/0026/0007) and is financed by the Carlos III Health Institute of Spain. Ten Spanish hospitals from various regions and healthcare levels are participating in the study, which together cover approximately 27,000 births a year. The coordinating center is Hospital 12 de Octubre of Madrid. The research groups involved in the study have experience in participating and coordinating relevant European and international projects.

These hospitals have beds with adjustable tilt for the women. Each of the centers is equipped with a remote SO_2_ and HR monitoring system (Masimo Patient SafetyNet®) for the newborns and have computer systems that provide 24-h access to the study’s computer platform to conduct the randomization and data entry into the electronic notebook. The mean number of researchers per center is 10, with a range of 2–20.

### Eligibility criteria for participants

All women who, at the start of labor, meet the inclusion criteria and none of the exclusion criteria (prerandomization criteria) and who have signed the consent form (see Additional file 2) will be included in the study and randomized using an ad hoc computer platform. In addition to the prerandomization exclusion criteria, we have established postrandomization criteria that will require withdrawal from the study, given that some events that occur during delivery may affect the newborn well-being. Table 1 lists the prerandomization inclusion and exclusion criteria, and Table 2 lists the postrandomization withdrawal criteria. Once 10 min have passed, and it is confirmed that none of the delivery- or neonate-related postrandomization withdrawal criteria have been met, all participating dyads will be included in the primary analysis.

**Table 1:** Prerandomization inclusion and exclusion criteria

*Prerandomization inclusion criteria*	
Women who meet the following criteria at the start of labor are considered candidates:	
(a) pregnant with a single fetus	
(b) monitored or partially monitored pregnancy (women who underwent ultrasonography at week 20 of their pregnancy but not during the first or third trimester or who did not undergo all trimester laboratory tests)	
(c) pregnancy proceeding normally or with mild disease (gestational diabetes treated exclusively with diet or arterial hypertension [with no diagnosis of preeclampsia] controlled with a single drug)	
(d) end of pregnancy at full term (37^+ 0^–41^+ 6^ gestational age)	
(e) temperature at start of labor ≤ 38 °C	
(f) presence of companion during the 2 h after delivery	
(g) mother’s wish for SSC	
*Prerandomization exclusion criteria*	
We excluded pregnant women before the randomization who met one or more of the following criteria:	
(a) consumption during pregnancy of tobacco, alcohol, drugs, or medication with sedative effects (opioids, antiepileptic drugs, antipsychotics, anxiolytics, hypnotics, antidepressants, and plants with sedative effects)	
(b) non-mild disease during pregnancy	
(c) prenatal diagnosis of chromosomal disorders or major malformation	
(d) prenatal diagnosis of intrauterine growth retardation with any degree of flow impairment, as well as an “abnormal” fetal size for gestational age (due to malformation, intrauterine infection, etc.)	
(e) lack of companion during the first 2 h postpartum

**Table 2:** Postrandomization criteria for withdrawal from the study

*Delivery related*	
(a) caesarean section or instrumental delivery (forceps, vacuum)	
(b) maternal fever > 38 °C	
(c) maternal hemodynamic instability (hypotension, tachycardia, impaired consciousness, poor perfusion, notable pallor) or any other type of ailment	
(d) umbilical cord prolapsed	
(e) signs of fetal “distress”, with fetal scalp pH < 7.25 or umbilical artery pH < 7.20	
(f) any other obstetric complication	
(g) use of sedatives or relaxants during or after delivery	
*Neonate related*	
(a) need for resuscitation measures	
(b) intrapartum-diagnosed major malformation	
(c) Apgar score ≤ 7 at 1, 5, or 10 min	
(d) presence of symptoms before 10 min of life	
(e) birth weight < 2300 g or > 4500 g	

### Interventions

Before starting patient enrollment, all participating centers have agreed upon the SSC procedure and the study intervention. Additionally, the coordinating center has conducted face-to-face training sessions in each of the hospitals.

**Fig. 1:**
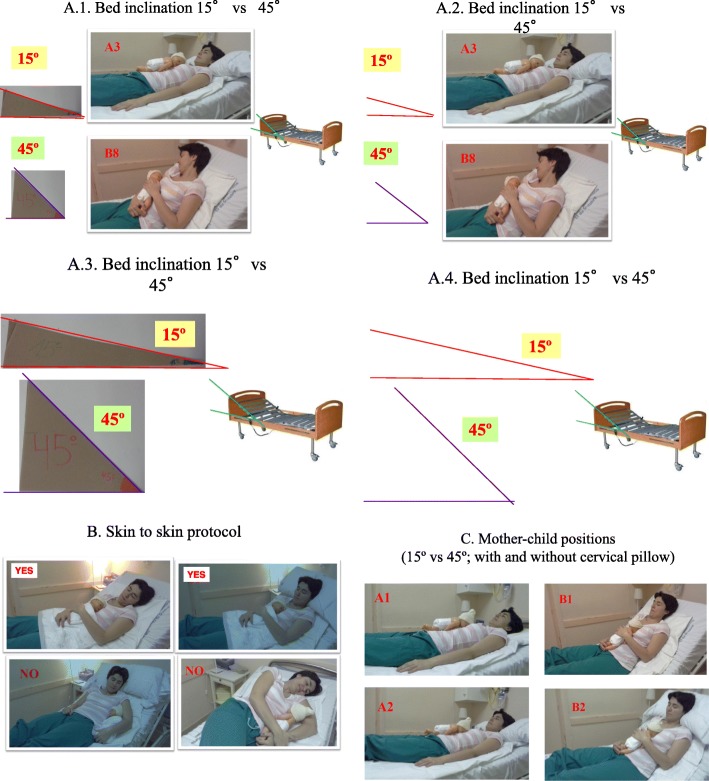
Models for mother’s and newborn’s positions for 15° and 45° inclination

When a possible case is identified, consent from the pregnant woman will be requested. Once the woman has agreed to participate, she is shown a number of photographs of how to perform SSC (Fig. 1; “Models for mother’s and newborn’s positions for 15° and 45° inclination”). The woman is told to record the moment(s) when the newborn latches on to the breast. The woman’s data are subsequently entered into the computer platform to proceed with the randomization. After the delivery, the researcher attending the birth will verify that the case does not meet any of the postrandomization withdrawal criteria. The pulse oximetry apparatus (noninvasive Radical-7 Signal Extraction PulseCO-Oximeter® monitor equipped with Masimo Rainbow SET® technology) is then plugged into the power outlet and to the data collection and is subsequently turned on. The pulse oximeter alarms have been established at 91% as the lower limit for SO_2_; the upper limit has not been set. For the HR, the lower limit alarm has been set to 111 bpm and the upper limit to 180 bpm.

During the first 10 min after delivery, with the newborn in SSC, the pulse oximeter sensor is attached (Masimo, LNCS® Series Neo-L adhesive sensor) on the right hand or wrist (preductal SO_2_). Considering the LNCS® sensor directions for use, the precision of the sensors is ± 3% for SO_2_ with or without movement and for the HR ± 3 bpm without movement and ± 5 bpm with movement. The saturation accuracy of the neonate was validated on adult volunteers and 1% was added to account for the properties of fetal hemoglobin. During these first 10 min after birth, the mother’s bed position is changed according to the corresponding randomization group (45° or 15° above the horizontal). To standardize the intervention, two cardboard models (15° and 45°) have been manufactured that exactly reproduce the angle (Additional file 1). During this period, diaper placement is the only intervention allowed.

At 10 min after birth, the sensor cable is attached to the pulse oximeter. After the connection, the pulse oximeter begins continuously recording the HR and SO_2_, storing the data and signal quality every 2 s. The pulse oximeter remains connected to a central station (Patient SafetyNet System, version V.4610, Masimo®) next to the newborn and mother for the 2 h of the study. The system transmits the data to the central station in real-time every second, displaying them on a monitor. The pulse oximeter’s alarms can be silenced and the screen light turned off so as to disturb the mother and newborn as little as possible. The alarms can be silenced in both the monitor and pulse oximeter. The pulse oximeter’s lighting can be reduced to a minimum to disturb the mother and newborn as little as possible. A practitioner is continuously monitoring the display and can respond to an alarm. As a safety mechanism, four locator-searchers (nurse, resident physician, staff physician, and supervising staff physician) provide alerts on the various alarms generated during the recording.

During the intervention period, the newborns remain at all times in SSC with their mother and will not undergo any procedure. If the newborn is separated from their mother for a reason other than those contemplated in the study (see alarm management protocol below), the recording will be stopped and the newborn will be removed from the study.

The mother may place a pillow behind her head but not behind her back and may choose her position in the bed, provided the assigned inclination angle is not changed (Fig. 1).

In those hospital centers in which the delivery and immediate postpartum do not take place in the same room, the transfer will be performed with the newborn in skin-to-skin contact, and the time of the transfer will be recorded. The monitor alarm, which will occur when disconnecting the pulse oximeter, will thereby be accounted for.

#### Action protocol in the event of an alarm or if a professional assessment is requested

**Fig. 2:**
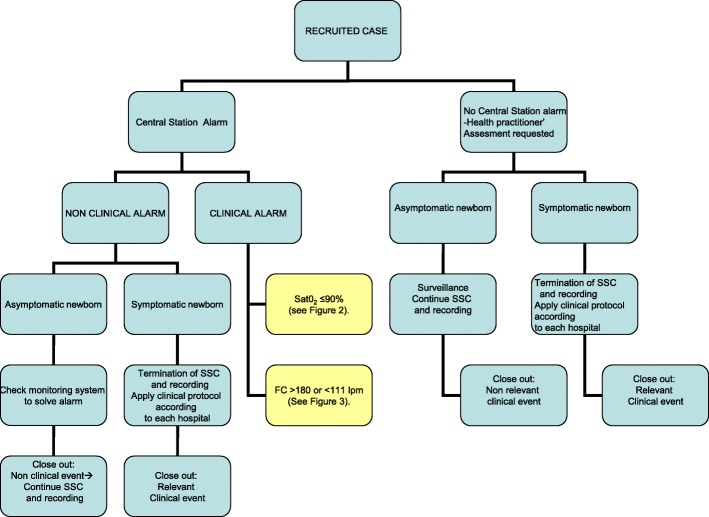
Action protocol when an alarm sounds or a professional assessment is requested

The protocol to be followed when an alarm sounds or when a practitioner is requested to come assess the newborn is shown in Fig. 2 (“Action protocol when an alarm sounds or a professional assessment is requested”).

Table 3 shows the data that will be collected when visiting the newborn for an assessment. The mother’s and newborn’s positions will be documented according to a series of model images created for each degree of inclination (Fig. 1; “Models for mother’s and newborn’s positions for 15° and 45° inclination”).

**Table 3:** Data recorded in case of alarm or if a professional assessment is requested

a. Number of alarms for this patient	
b. SO_2_, HR, and time	
c. Minimum SO_2_ during the episode and corresponding HR	
d. Minimum or maximum HR as appropriate and corresponding SO_2_	
e. Time to once again reach an SO_2_ > 90%	
f. Position of the mother and child	
g. Probable corresponding factor	
h. Child’s clinical situation	
i. If it entails terminating the procedure	
j. Possibility of airway obstruction in the child	

The practitioner will classify the alarm as clinical or nonclinical depending on whether the events that justify these alarms are clinical (CEs) or nonclinical events (NCEs), respectively. The CEs can, in turn, be clinically relevant (CREs) or nonclinically relevant events (NCREs).

NCEs do not entail that the intervention is terminated and can occur due to excessive ambient light, problems with the pulse oximeter’s connection cable or sensor, low battery levels, interference, and lack of system connectivity or availability.

**Fig. 3:**
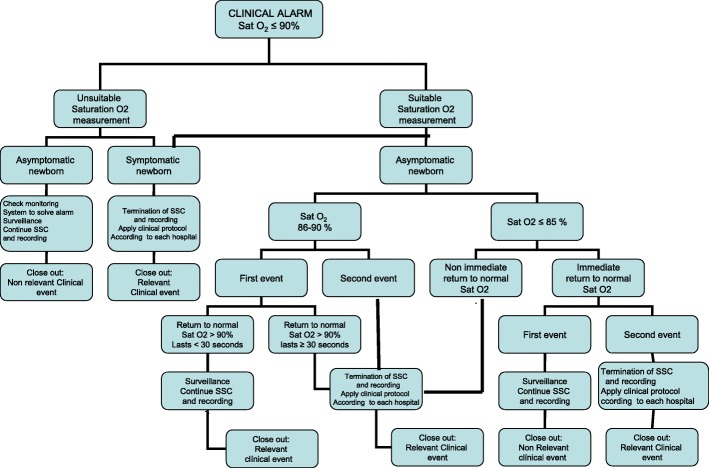
Action protocol when a SO_2_ < 91% clinical alarm goes off

**Fig. 4:**
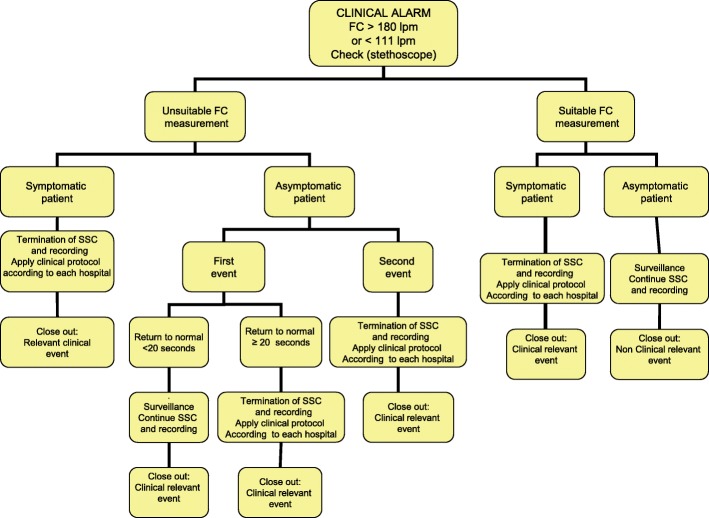
Action protocol when a HR > 180 or < 111 bpm clinical alarm sounds

The CEs include an SO_2_ < 91%, a HR > 180 bpm or < 111 bpm, and a newborn with symptoms. The protocol to be followed when clinical alarm sounds is shown in Figs. 3 and 4 (Fig. 3, action protocol when a SO_2_ < 91% clinical alarm goes off; Fig. 4, action protocol when a HR > 180 or < 111 bpm clinical alarm sounds). A CE is considered nonclinically relevant (NCRE) if the newborn is asymptomatic and the event can be explained by a technical problem (insufficient signal capture) or by the newborn’s condition at that moment (crying, newborn handling by the mother, movement, etc.). NCREs do not entail the termination of the intervention.

If the CE cannot be explained by any of the above causes, the CE will be considered a CRE depending on whether it occurs for the first or second time, the magnitude of the desaturation, additional heart rate impairment, or if the newborn is symptomatic. A CRE entails the termination of the intervention.

Investigators at the coordinating center will review the compliance with the intervention protocols. If noncompliance is detected, the head of each participating center will be contacted, and the deviations from the protocol will be discussed.

### Outcomes

The main outcome collected in both intervention groups will be the proportion of healthy newborns who undergo at least 1 episode of SO_2_ ≤ 90%.

The following secondary outcomes will also be recorded:

1. The mean SO_2_ level and HR in the first hour, in the second hour, and for both hours combined.
2. The proportion of newborns who undergo at least one episode of SO_2_ ≤ 85%.
3. The proportion of SSC interruption due to a medical indication.
4. The number of HR episodes < 111 bpm and > 180 bpm.
5. The mother’s posture (right lateral decubitus, left lateral decubitus, or supine) and that of her child (right lateral decubitus, left lateral decubitus, supine, or prone) at the time of the episode of SO_2_ ≤ 90% or HR < 111 bpm.

### Participant timeline

**Fig. 5:**
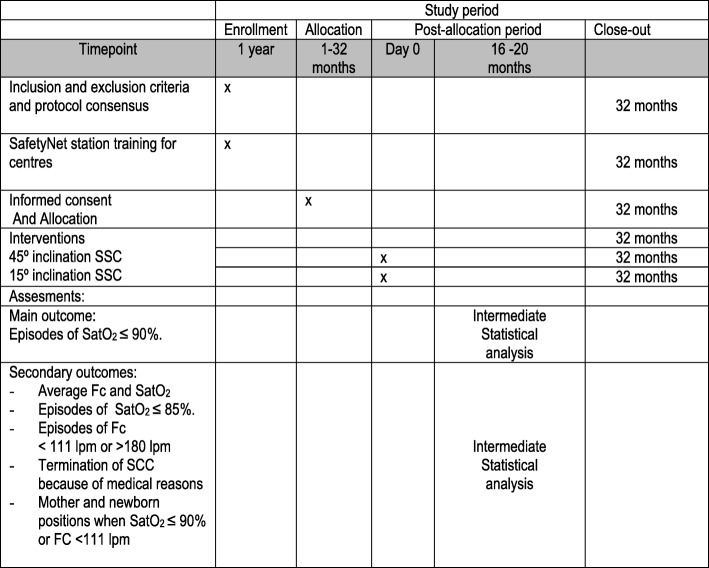
SPIRIT. Schedule of enrollment, interventions, and assessments

The participant timeline is shown in Fig. 5.

### Sample size and recruitment

We conducted an observational pilot study in the coordinating center. In this study, the mothers performed SSC with their child in the first 2 h postpartum, and there was no intervention on the maternal position adopted. The mothers could choose their position, partially sitting up with an angle between 15° and 50°. We observed that approximately one of every ten healthy newborns had an episode of SO_2_ ≤ 90%.

To calculate the study’s sample size, we predetermined that, with the bed in the 15° and 45° positions, the rate of episodes could be 9% and 6%, respectively. To estimate a reduction of a third in the number of newborns with episodes of SO_2_ ≤ 90% (with a 95% confidence level and a power of 80%), we calculated that we would need to include 1275 newborns in each group, which represented a total of 2550 newborns. In the pilot study, we observed that approximately 50% of the initially included cases had to be excluded due to established postrandomization criteria (see Table 2). Therefore, the estimated number of dyads to randomize would be 5100. We deemed it necessary to include 15% more participants for operational reasons and to conduct an intermediate analysis. The final figure for participants would therefore be 5866.

This number would be easily recruitable among all the centers involved in the study. The recruitment will last 32 months.

### Allocation and blinding

The randomization was conducted using permuted blocks. To this end, we employed a shared computer platform. Each center was assigned a block, and the patients were randomized one after another according to the random numbers contained in the corresponding block. Each center’s block size was created after an initial calculation of the number of mother/newborn dyads the center could recruit over the course of a month (the block sizes ranged from 193 to 1076 cases). The patients will be randomized at the start of labor. Once the informed consent form is signed, the researcher who obtained the consent will access the shared computer platform with the center’s specific login. The platform will then take the researcher directly to their block of random numbers and show them the mother’s degree of inclination. Beforehand, the researcher must answer a series of questions to confirm that the dyad meets all study inclusion criteria and none of the exclusion criteria. The center’s researcher is not allowed to know the block of numbers they are given nor the sequence of numbers. The recorded pulse oximetry data (SO_2_ and HR) will be extracted and analyzed in the coordinating center by researchers who will be blinded to the mother’s sitting angle in bed.

### Data collection, management, and monitoring

In each case, a number of characteristics related to the mother, delivery, and newborn will be recorded (Table 4). These data will be inserted a posteriori in the electronic case report form created for the study and will be accessible for those responsible for the study in the coordinating center. The recorded information corresponding to the SO_2_ and HR for each case will be electronically downloaded through various report formats, which in turn will be submitted to the coordinating center by e-mail. The coordinating center has the appropriate software for reading and analyzing the recorded pulse oximetry data.

**Table 4:** Demographic data to be recorded in each case

- *Mother-related*: age, country of origin, medical history of interest, parity, desire to breastfeed, increase in weight during pregnancy, body mass index, bra size (including cup size), a history of children who died, and medication consumed during the 72 h prior to delivery	
- *Delivery-related*: date and time, duration, time of completion, spontaneous or induced, delivery position, epidural anesthesia, time of epidural, time the water broke, nuchal cord, episiotomy, artery and umbilical vein blood gas test, maternal medication during labor and 2 h afterwards.	
- *Neonate-related*: sex, weight, gestational age, Apgar score, cord pH, presence of crying at birth, and time of the first breast latch	

The implementation of post-randomization exclusion criteria will be evaluated in a reliability study. Two investigators will independently assess the criteria in a sub-sample of study participants. Reliability and agreement statistics will be reported.

### Statistical methods

As we estimate a relevant proportion of estimated postrandomization exclusions, we will perform a modified intention to treat analysis.

The study will employ a two-tailed significance level of 5%. The confidence intervals will be 95%. The main assessment criterion is the presence of an episode of SO_2_ ≤ 90%. We will perform an unadjusted analysis of the intervention’s effect with chi-squared test.

The distribution of the baseline prognostic variables will be analyzed with the mean and standard deviation for the continuous variables (we expect a normal distribution) and with numerators/denominators and percentages for the categorical variables. *P* values will not be included, but we will indicate the variables that have a strong association that could have a confounding effect—even if the distributions in the two study groups are not very different—and the variables with large differences between the groups that could have a confounding effect, even those with a poor association. Stratified analysis and logistic regression modeling will be performed on the primary outcome to take into account covariates (i.e., exploring the possible effect of modification of breast feeding, adjusting for relevant covariates, etc.). The strategies for missing-data imputation and the subgroup analysis will be documented a priori.

The statistical analysis will be conducted using the software environment R [28].

An independent committee, which will include an independent biostatistician, will review the results of the interim analysis.

The study will be halted if differences in the main outcome show a *p* value below 0.001 to protect the overall type I error of the trial. The study will also be interrupted on safety grounds due to any other results that show a negative effect on the child or mother considered relevant by the committee.

### Ethical aspects

Problems were detected after an initial pilot trial. A meeting was held with all centers from which emerged proposals for changing the initial trial protocol. These changes were presented to the coordinating center’s Clinical Research Ethics Committee (EC), who approved the changes (2015-06-30), which were then sent to the ethics committees of the participating hospitals, who in turn approved the changes.

One of the required inclusion criteria is that the mother must have a companion during the first 2 h postpartum, given that the episodes have been more frequently reported when the mothers were not accompanied during this period. There is currently no recommendation on the ideal position for the mother and child immediately after delivery. Therefore, neither of the two positions in the study differ from any standard nor change the established risk. In current standard practice, newborns in SSC are not monitored.

The principles of the Declaration of Helsinki will be respected during the project’s implementation. All documentation generated by the study shall be treated, communicated, and transferred in accordance with the requirements laid out in the Spanish Organic Law 15/1999 of December 13 on the Protection of Personal Data. Documentation confidentiality will be ensured. The study protocol was submitted for approval to the Clinical Research Ethics Committee of Hospital 12 de Octubre and those of the other participating centers. A single informed consent (Additional file 2) form was prepared for the women who decided to participate in the study. If a woman decides to withdraw her consent for participating in the study, no new datum will be added, and she may request the erasing of her already recorded data.

Each of the participating centers has a researcher responsible for guarding and updating all information generated by the trial in their hospital, both for hardcopy and electronically generated information. At the coordinating center, this researcher will be responsible not only for the data produced in their hospital but also for that submitted by each center regarding the reports on the records of the enrolled cases.

The results of this study will be published in pediatric journals in the top 10% in terms of impact factor. Furthermore, all information and results from the trial will be presented in national and international scientific meetings. Once the study has concluded, the results will be recorded on the Clinicaltrials.gov website in which it is registered.

## Discussion

This randomized clinical trial seeks to show whether the mother’s sitting position at 45° or 15° above the horizontal plane influences the full-term newborn’s cardiopulmonary stability without complications while in SSC during the first 2 h postpartum. The results of this study will provide information on the newborns’ risk of ALTEs or sudden death while this procedure is performed in the abovementioned time interval.

All etiological hypotheses consider that newborns (in the first 2 h after delivery) are susceptible to hypoxic episodes, either from a central origin or by obstructive problems [12–14, 16, 17, 19–21, 25–27]. Except for the study by Davanzo et al. [29], no prospective studies in the scientific literature have assessed the circumstances that could contribute to the onset of hypoxic episodes in the first 2 h of life.

This study, which uses the methodology of a clinical trial, will be the first to assess whether the mother’s bed incline in the immediate postpartum could influence the onset of desaturation episodes and/or changes in the HR in the newborn. In the SSC position, the newborn is usually in decubitus prone on their mother. It is easy to assume that the more reclined the mother, the greater the possibility of compromising the newborn’s breathing, even more so during the first 2 h of life, a critical period for adapting to extrauterine life. This study will also assess the role of breastfeeding during SSC in the onset of these events.

This clinical trial is the first to continuously monitor SO_2_ levels and HR during the first 2 h postpartum in full-term newborns with no complications and delivered vaginally who are in SSC with their mother.

Our understanding of the progression of SO_2_ in newborns in SSC is scarce. A number of studies have examined the SO_2_ range of full-term healthy newborns, but only during the first minutes of life. It has been observed that newborns have low SO_2_ levels that increase slowly, without generally reaching the 90% level after 5 min [30–33]. Other observations have noted a significant gradient between the SO_2_ of preductal oxygen (higher) and postductal oxygen during the first 15 min after delivery [32, 33]. There is no uniformity in terms of the time needed to achieve an SO_2_ of 90% between newborns born by caesarean section and those born vaginally [31, 33–35]. In terms of the progression of SO_2_ in full-term newborns in SSC, our understanding is much lower. Dawson [36] reported that a mean of 7.9 min (interquartile range, 5.0–10.0 min) is required to achieve an SO_2_ > 90%. Takahashi [37] studied the progression of SO_2_ and HR for 30 min in two groups of newborns. In the first group, SSC was started during the first 5 min after delivery. In the other group, SSC was started after 5 min or more. The group that started SSC earlier achieved HR stability sooner (between 120 and 160 bpm), but there were no differences in terms of SO_2_ stability (readings between 92% and 96%).

Other factors that contribute to the relevance of this study are that it focuses on a clinical problem of considerable significance for the health of the newborn who experiences this condition, one that has been reported in practically all developed countries. In addition, currently available information on this problem is very scarce [14, 15, 18, 19, 24–26]. This is the first time this problem has been approached with a clinical trial design, with a multicenter approach and a considerable sample size. Another point of interest is that these potential risk situations will be documented with model images.

Based on the study results, a preventive strategy for these episodes of sudden death and ALTEs could be designed. If keeping the mothers in a more upright position is associated with a lower rate of desaturation in the newborn, the change in maternal position could act as a protective factor for ALTEs and sudden deaths in the first 2 h.

The study is multicenter, which could entail certain variability in a number of the study procedures despite previous agreements, which could represent a limitation. Thus, in the hospitals in which the delivery and postpartum take place in different rooms, the pulse oximeter will be disconnected while the mother and child are transferred, and there will be a loss of data during this time interval. The SSC procedure itself or the method for achieving proper bed inclination might not be uniform. Lastly, the clinical practice of the various centers regarding the clamping of the umbilical cord (early or late clamping, umbilical cord milking) varies. Another limitation consists of the fact that the study will not have the services of a contract research organization (CRO). The coordinating center’s researchers will be responsible for monitoring the proper application of the study protocol.

The study results may not be applicable to newborn groups with different characteristics but who are still candidates for SSC (late-preterm newborns, newborns with disease during the pregnancy, and/or delivery that does not contraindicate SSC).

One of the study risks is that it can lead to the monitoring of all newborns during their time in SSC and potentially to separating many newborns from their mothers. This is not the study’s objective, which seeks only to observe the effect of the bed position.

The study is focused on analyzing what occurs during the first 2 h after delivery. It would be interesting to extend the analysis time to the first 24 h of life while the mother and child are in SSC. It would also be important to determine whether the degree of inclination could influence the mother’s recovery and state of health during the immediate postpartum.

Even if the results of this study can shed some light on our understanding of these events, they will not preclude medical personnel from continuing to give mothers and their companions the recommendations that various experts have indicated in terms of safely implementing SSC [12, 13, 15, 16, 20–22, 24, 27, 29, 38, 39].

### Trial status

The trial has completed the planning and pilot phases and is currently enrolling participants. The first patient was recruited in November 2015.

## Additional files

Additional file 1: Corresponds to List of SPIRIT confirmation. (DOCX 20 kb)

Additional file 2: Corresponds to Informed Consent. (DOCX 51 kb)

## Abbreviations

ALTE: Apparent life-threatening event
Bpm: Beats per minute
CE: Clinical event
CRE: Clinically relevant event
HR: Heart rate
NCE: Nonclinical event
NCRE: Nonclinically relevant event
SAMID: SpanishNetwork, Spanish Collaborative Maternal and Child Health and Development Research Network (*Red Española de Investigación en Salud Materno Infantil y del Desarrollo*)
SO_2,_: Oxygen saturation
SSC: Skin-to-skin contact
