# Supplementary material for: What is the impact of mother’s bed incline on episodes of decreased oxygen saturation in healthy newborns in skin-to-skin contact after delivery: Study protocol for a randomized controlled trial
Source: Trials. 2019 Mar 20;20:179. doi: 10.1186/s13063-019-3256-0 (PMC6427856; doi:10.1186/s13063-019-3256-0)
Supplement: Supplementary file 2 — Corresponds to Informed Consent. (DOCX 51 kb) [file 13063_2019_3256_MOESM2_ESM.docx]

**Additional file 2. INFORMED CONSENT for participation in the study.**

**“Episodes of decreased oxygen saturation in newborns in skin-to-skin contact in relation to the maternal position after delivery. Randomized clinical trial”**

We are writing you to inform you of the possibility of participating (you and your child) in the following study. Participation in this study is voluntary. If you decide to participate, you may withdraw your consent at any time without affecting your relationship with the medical personnel or the care you will receive throughout the process of delivery.

The aim of this study is to understand how the quantity of oxygen in the blood of newborns changes over the course of the first 2 hours after delivery while in skin-to-skin contact with their mother (that is, the newborn is placed face down on their mother’s chest or abdomen). Skin-to-skin care has proven advantages for newborns and their mothers, but little is known about certain aspects of the newborn’s body functions. You will be shown a number of images that will help you better understand how this procedure is performed. If you give us your consent to participate in the study, you will be randomly assigned to 1 of 2 possible positions in which you will remain for the first 2 hours after delivery. The 2 positions are with the head of the bed raised, either to 15 degrees or 45 degrees above the horizontal. Ten minutes after your child is born, the bed you are in at the time (either the bed where the delivery took place or the bed you will be in for the 2 hours after delivery) will be set to the assigned position. If you wish, a pillow may be placed behind your head but not behind your back, because this could change the angle at which you and your child are seated.

During the study, the early skin-to-skin contact protocol will be followed. This protocol is routinely applied in this hospital center. Participation in this study entails the following changes to the aforementioned protocol. Ten minutes after birth, a cable will be placed on your child. One end of the cable has a band that will be wrapped around your child’s right hand or wrist. The other end of the cable will be connected to a machine (a pulse oximeter), which has a screen. The band that will be placed on the hand has a red light that detects your child’s heartbeat and quantity of oxygen in the blood at any moment. The placement of this apparatus and the red light it emits causes no pain. The only thing your child will feel is a little pressure on the hand. The detected data will be reflected on the machine’s screen. The data will also be sent to a central monitor located in the Department of Neonatology. There will always be a healthcare practitioner (neonatal nurse or neonatologist) monitoring this information at all times. If any of the apparatus’ alarms goes off, either due to abnormal oxygen levels or changes in your baby’s heart rate, these will immediately be reflected on the central monitor. One of the clinicians will immediately assess your child and act accordingly. So that neither the noise nor the light of the pulse oximeter interfere with your relationship with your child while you are in skin-to-skin contact, the alarm volume will be lowered as much as possible and the light intensity decreased on the machine’s screen.

There will be no changes in your care, the personnel who will care for you and your child or in the action protocols in the event either of you require them. Neither you nor your child will be subjected to any additional risk to your health. The only expected inconvenience of your participation in the study is that you will have to remain for 2 hours without changing the angle of your bed. After 2 hours, this might be a little uncomfortable.

The study will only include healthy newborns with a gestational age at birth of 37 weeks or more, who require no resuscitation of any type at the time of birth. It might happen that you decide to participate in the study and then are unable to participate because you and/or your child need medical care.

The data and information recorded by the researcher and by the pulse oximeter will go on to be part of a database for later analysis. At all times, this documentation will be treated, communicated and transferred in accordance with the Spanish Organic Law 15/1999 of December 13 on the protection of personal data. Documentation confidentiality will be ensured. If you decide to withdraw your consent for participating in the study, no new data will be added, and you may request erasure of the already recorded data.

Thank you for your help.

**If you need more information or clarification, don’t hesitate to ask.**

**STATEMENTS AND SIGNATURES**

I declare that...

- I have been clearly informed of the nature and risks of the study in question.

- I am satisfied with the information I have received. I have asked all the questions I believed relevant, and my questions have been clarified.

- I therefore give my consent to participate in the study.

- I understand that participation in the study is voluntary.

- I have been informed of the possibility of cancelling this consent at any time, without having to provide an explanation and without it affecting the care that my child and I will need. I agree to sign this refusal if this should happen.

Date: __/__/____

***Mother’s signature. Signature of the individual who provided the information. Researcher’s signature***.

**Name of the legal representative: _____________________________________ Nat ID:______________**

As the mother, I authorize the participation in the study in question.

**Name of the individual who provided the information: ______________________________________________________**

**Researcher’s name: ______________________________________________________________**

**REFUSAL / CANCELLATION OF CONSENT Date: __/__/____**

After being informed of the nature and risks of the proposed study, I declare freely and consciously my **REFUSAL/CANCELLATION** to participate in the study in question.

***Mother’s signature. Signature of the individual who provided the information. Researcher’s signature***.

**Name of the legal representative: _____________________________________ Nat ID:______________**

As the mother, I decline to participate in the study in question.

**Name of the individual who provided the information: ______________________________________________________**

**Researcher’s name: ______________________________________________________________**
